# Invasive meningococcal disease due to a non-capsulated *Neisseria meningitidis* strain in a patient with IgG4-related disease

**DOI:** 10.1186/s12879-018-3064-2

**Published:** 2018-04-02

**Authors:** Shun Kurose, Kyoko Onozawa, Hiroshi Yoshikawa, Kenichiro Yaita, Hideyuki Takahashi, Nobuyuki Shimono, Yoji Nagasaki

**Affiliations:** 1 Division of Infectious Diseases, Fukuoka City Hospital, 13-1 Yoshizuka-Honmachi, Hakata-ku, Fukuoka, 812-0046 Japan; 2 Department of Ophthalmology, Graduate School of Medical Science at Kyushu University, Fukuoka, Japan; 3 Division of Infection Control and Prevention, Kurume University Hospital, Kurume, Japan; 4 Department of Bacteriology I, National Institute of Infectious Diseases, Tokyo, Japan; 5 Center for the Study of Global Infection, Kyushu University Hospital, Fukuoka, Japan

**Keywords:** Invasive meningococcal disease, Non-capsulated *Neisseria meningitidis*, IgG4-related disease, Hypocomplementemia

## Abstract

**Background:**

Invasive Meningococcal Disease (IMD) is a rare and critical disease in Japan. Most of these cases are caused by capsulated *Neisseria meningitidis* strains. Non-capsulated (non-typable) strains are considered relatively low-pathogenic and can colonize in the nasopharynx of healthy children and young adults. As far as could be ascertained, only twelve IMD cases due to non-capsulated strains have been reported in the literature. No clear risk factors could be identified in a literature review (unknown or immunocompetent, seven cases; C6 deficiency, three cases).

**Case presentation:**

We report a Japanese male taxi driver with bacteremia and meningitis due to non-capsulated *N. meningitidis.* He had a fever and shaking chills. Ceftriaxone was administered, and the patient finally recovered. During the clinical course, relative adrenal insufficiency occurred and was treated with hydrocortisone. A hidden co-morbidity, immunoglobulin G4 (IgG4)-related disease, was revealed in the past surgical history (a resection of bilateral orbital tumors), which included symptoms (swelling lachrymal glands and lymph nodes), elevated IgG4, immunoglobulin E, and hypocomplementemia. He recovered finally and no recurrence was observed.

**Conclusions:**

Our IMD case is the first reported in Japan, where IMD is not considered pandemic. The patient had a history of IgG4-related disease, although we could not establish a clear relationship between the patient’s IMD and co-morbidity. A collection of further clinical cases might establish the risk factors and characteristics of IMD that could be caused by this neglected pathogen, non-capsulated *N. meningitidis*.

## Background

Invasive Meningococcal Disease (IMD) is a critical disease caused by *Neisseria meningitidis*. In Japan, IMD was listed as a notifiable disease in April 2013. Approximately 60 cases were reported to Japanese national surveillance from April 2013 to December 2014 [1].

*N. meningitidis* causing IMD usually have the capsule [2]. Encapsulated bacteria are resistant to humoral immunity and have a tendency to disseminate hematogenously. On the other hand, non-capsulated (non-typable) *N. meningitidis* strains are not resistant to opsonization and seldom cause invasive infection in healthy humans. Therefore, a search of the literature reveals only a small number of case reports of IMD due to non-capsulated *N. meningitidis*.

As far as we could ascertain, this is the first Japanese case of IMD due to non-capsulated *N. meningitidis*. The patient had an underlying diagnosis of immunoglobulin G4 (IgG4)-related disease. By describing this case and the review of the past literature, we discuss the neglected pathogen, non- capsulated *N. meningitidis*. And we consider the possible relationship between IMD and IgG4-related disease.

## Case presentation

A 51-year-old male taxi driver was admitted to Fukuoka City hospital with high fever (40 °C) and a shaking chill despite his use of over-the-counter antipyretics. He was transferred to the emergency department about 14 h after the symptoms onset. His medical history included bronchial asthma, pneumonia, and a surgical procedure for the resection of bilateral orbital tumors at 40 years of age. The patient had neither a history of contact with sick persons nor of any travel abroad. His body height was 160 cm and his body weight was 52 kg.

**Fig. 1:**
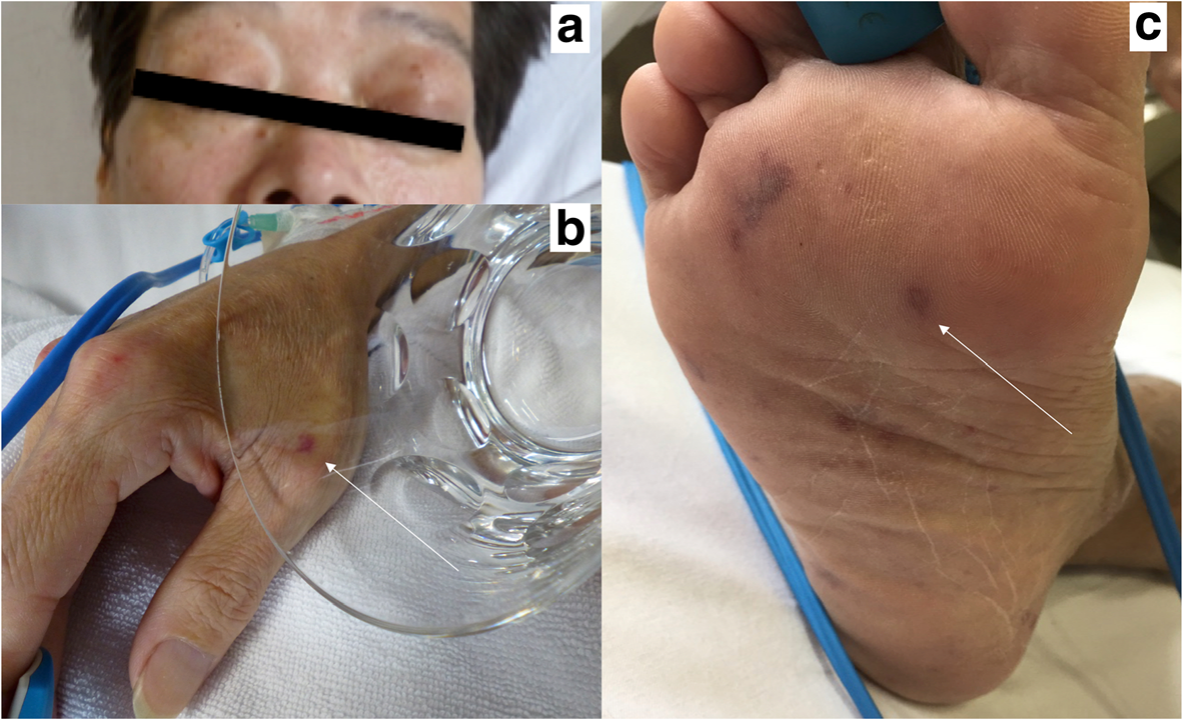
A swelling of bilateral eyelids (**a**) and purpura and petechiae on the limbs (**b**, **c**)

**Fig. 2:**
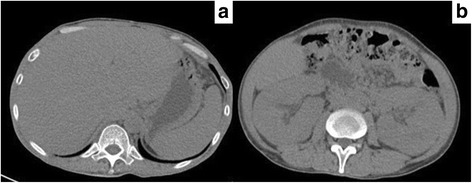
The enlargement of liver and bilateral kidneys (**a**, **b**) detected by using computed tomography

At the time of admission, his vital signs were as follows: blood pressure, 108/74 mmHg; heart rate, 90 beats/min; respiratory rate, 40 breaths/min; body temperature, 40.1 °C; and Glasgow Coma Scale, E4V4M6 (total 14/15). Upon physical examination, a swelling of the bilateral eyelids (Fig. 1a) and petechiae were detected on the conjunctiva. Some dental caries, purpura and petechiae were noted on the limbs (Fig. 1b, c), and 1–2 cm sized swollen lymph nodes noted at submandibular and inguinal areas were remarkable. Neck stiffness was not detected. Laboratory data on admission revealed high inflammation (white blood cell 13,500 μL, and c-reactive protein 12.29 mg/dL), normocytic anemia (Hemoglobin 8.7 g/dL), high protein and low albumin levels (total protein 12.3 g/dL and albumin 2 g/dL), renal failure (serum creatinine 4.52 mg/dL), hyperkalemia (6.12 mEq/L), proteinuria and hematuria (Table 1). Computed tomography showed enlargement of the liver and bilateral kidneys (Fig. 2a, b) in addition to swelling of the cervical, supraclavicular, axillary, mediastinal, and celiac lymphadenopathy.

**Table 1:** The laboratory data of the patient

Hematology			Chemistry			Serology		
WBC	13,500	/μl	TP	12.3	g/dl	IgG	7392	mg/dl
Neutro	84.5	%	Alb	2	g/dl	IgG4	3200	mg/dl
Lymph	9.6	%	Glu	69	mg/dl	IgA	78	IU/ml
Hgb	8.7	g/dl	T-Bil	0.37	mg/dl	ANA	< × 40	
HCT	25.9	%	AST	13	IU/l	SS-A	7.8	U/ml
PLT	15.4	10^4^/μl	ALT	26	IU/l	SS-B	≦7.0	U/ml
Coagulation			LDH	282	IU/l	CH50	<12.0	U/ml
PT %	40	%	CK	123	IU/l	C3	37	mg/dl
APTT	48.6	sec	BUN	46.3	mg/dl	C4	7	mg/dl
FDP	10.5	μg/ml	Cre	4.52	mg/dl	ACTH	66.9	pg/ml
Urinalysis			Na	125	mEq/l	Cortisol	16.7	μg/dl
Protein	(1+)		K	6.12	mEq/l	sIL-2R	5390	U/ml
Blood	(1+)		Cl	102	mEq/l	Procalcitonin	> 10	ng/mL
Bence-Jones protein	(−)		CRP	12.29	mg/dl			
β2-microglobrin	103,000	μg/l

**Fig. 3:**
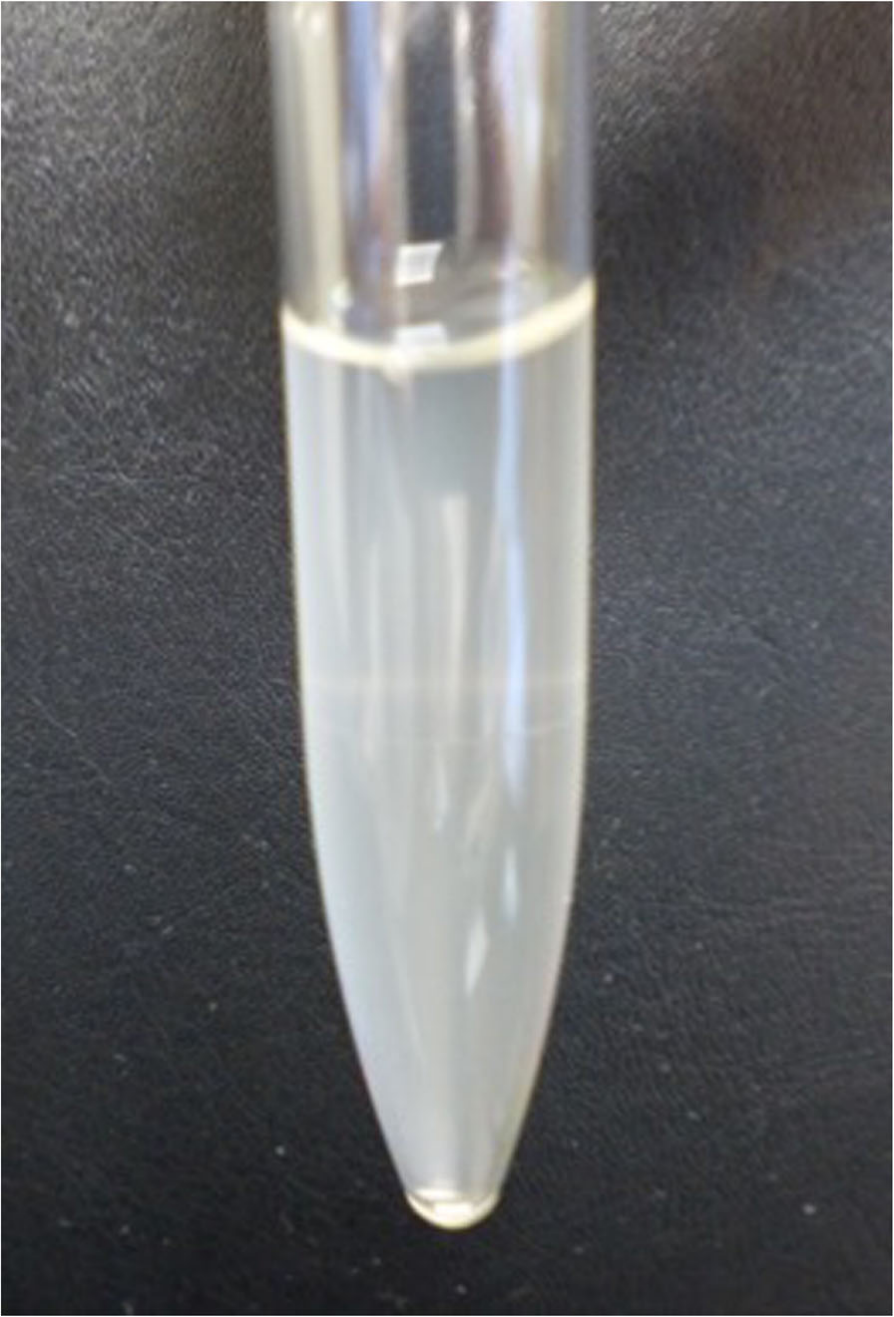
A lumbar puncture was performed and the turbid spinal fluid was collected

**Fig. 4:**
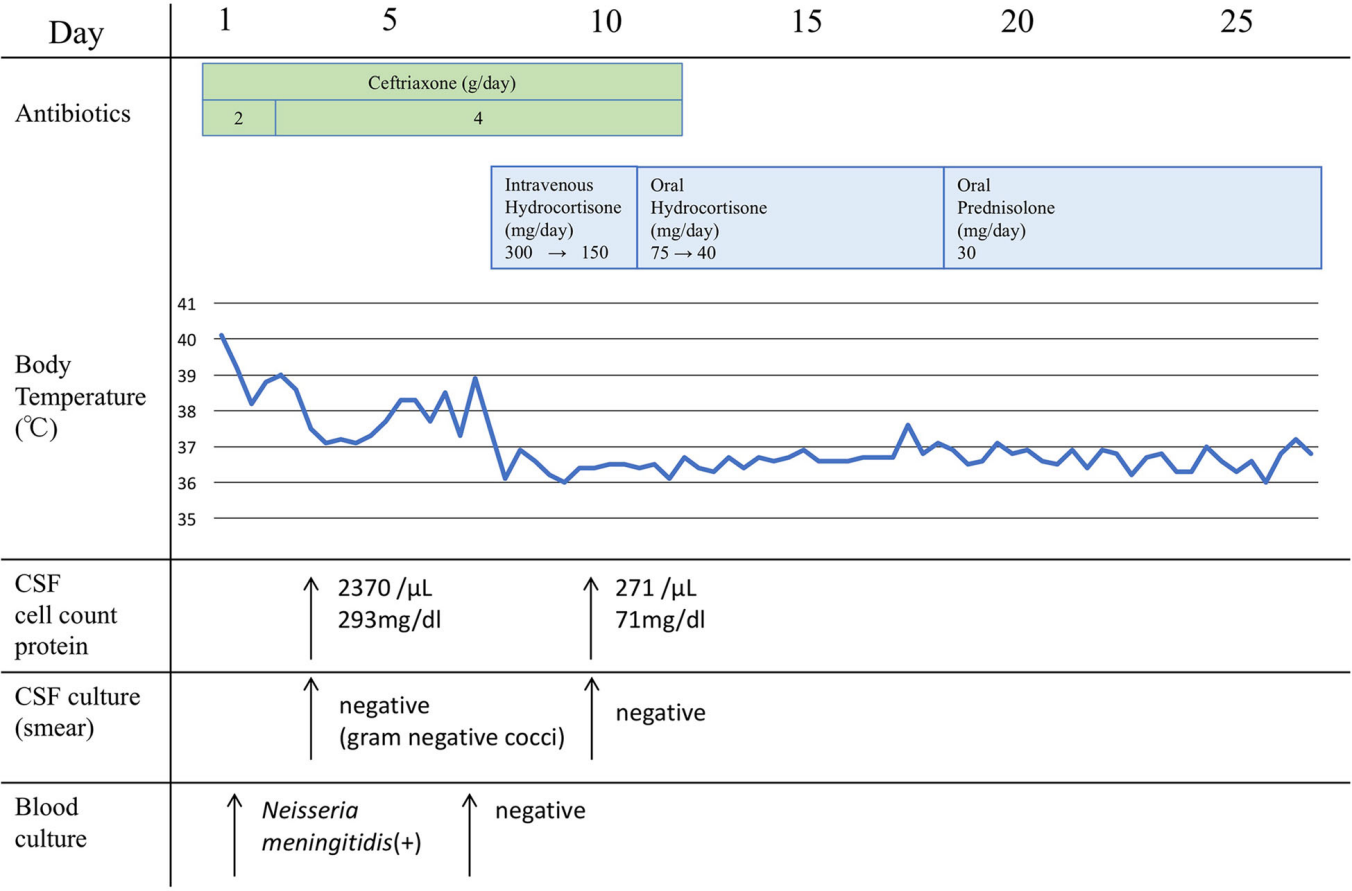
Clinical course of the case. The patient was managed successfully with antibiotic and hydrocortisone and discharged on day 25

Severe infectious disease was suspected, and we started ceftriaxone 1 g intravenously every 12 h after drawing a blood culture. However, purpura spread rapidly and the progression of drowsiness and fever was sustained on the 2nd hospital day. We performed a lumbar puncture and the turbid spinal fluid analysis revealed meningeal inflammation (white blood cell count, 2370 /fields; protein, 293 mg/dL; and glucose, 16 mg/dL), which was compatible with bacterial meningitis (Fig. 3). Gram-staining of the spinal fluid showed gram-negative diplococci. The blood culture also became positive for gram-negative diplococci. We strongly suspected that the septic status of the patient’s meningitis and bacteremia was due to *N. meningitidis*. We increased the dose of ceftriaxone to 2 g intravenously every 12 h. The patient’s general status and consciousness then improved and his fever subsided. On the 5th hospital day, the patient’s fever (38 °C) re-appeared with hypotension, hyponatremia and eosinophilia, which led to a suspicion of relative adrenal insufficiency due to meningococcemia. We started a daily intravenous administration of 300 mg hydrocortisone on the 7th hospital day, and tapered the regimen to a daily oral administration of hydrocortisone on the 11th hospital day. Ceftriaxone was stopped on the 12th hospital day. No recurrence of infection was observed and he was discharged on the 25th hospital day. The clinical course is summarized in Fig. 4.

We investigated the patient’s co-morbidities and performed microbiological analysis of the strain. His past surgical history (bilateral orbital tumors), his symptoms (swelling lachrymal glands and lymph nodes), the elevated levels of IgG4 (3200 mg/dL) and immunoglobulin E (304 IU/ml), and hypocomplementemia (C3 37 mg/dL, C4 7 mg/dL, and CH50 < 12.0 U/ml) were the characteristics of Mikulicz’s disease included in IgG4-related disease. Furthermore, a biopsy of the patient’s lymph nodes confirmed the presence of IgG4-related disease (IgG4/IgG index 100%). We prescribed oral prednisolone 30 mg a day on the 19th hospital day. In addition, the strain of *N. meningitidis* was identified as non-typable. The sequence type (ST) was identified as ST-11448, which belongs to ST-23 (ST-23 complex), the most common ST in Japan [3]. Moreover, we also analyzed the *N. meningitidis* strain according to PorA and FetA typing, which has a higher resolution power than that of ST, and identified that the PorA VR1, PorA VR2 and FetA were 5, 2–82, and F4–1, respectively. These results suggested that this *N. meningitidis* strain was a non-capsulated derivative of the domestic ST-23 strain. (See Discussion and conclusion).

## Discussion and conclusion

As far as we could ascertain, this is the first Japanese case among IMD cases due to non-typable *N. meningitidis*. This is also the first case of *N. meningitidis* causing IMD in a patient with IgG4-related disease.

The mechanism of *N. meningitidis* infection relies on three factors to avoid the human immune response system: the formation of a capsule, the production of IgA protease, and molecular mimicry. Among these factors, the formation of a capsule contributes the most to the progression of IMD [4]. A capsule can prohibit opsonophagocytosis in the human body. Twelve serogroups of *N. meningitidis* (A, B, C, Y, W, X, Z, 29E, H, I, J, L) were divided according to the antigenicity. Among these serogroups, the five (A, B, C, Y, W) make up the majority of IMD causative pathogens, for which vaccines are available [5]. On the other hand, non-typable strains are considered to be of low-pathogenicity [6].

Only 12 IMD cases due to non-typable strains could be found in a literature review by using PubMed and Ichushi (the Japanese database for medical literature and conference proceedings) databases (Table 2) [4, 7–12]. Although the risk factors of most cases are unknown, three C6 deficiency (hypocomplementemia) cases are described in the literature [7, 12]. Hypocomplementemia is an established risk factor of IMD [13]; however, there has been no report of the relationship between hypocomplementemia and IMD due to non-typable bacteria. Opsonophagocytosis with antibodies and complements is thought to prevent the invasion of non-capsulated bacteria [13].

**Table 2:** The previous reports of invasive meningococcal disease cases due to non-typable *Neisseria meningitidis*

Case	Age	Sex	Risk Factor	Result of Culture	Outcome
Hummell DS, et al. 1987 [7]	0	M	C6 deficiency	CSF: positive Blood:negative	Survive
	0	M	C6 deficiency	CSF: positive Blood:negative	Survive
Vogel U, et al. 2004 [4]	42	–	ALL, GVHD	Blood: positive	Survive
Hoang LM, et al. 2005 [9]	13	F	Immunocompetent	CSF: positive, Blood: negative	Death
Findlow H, et al. 2007 [8]	12	M	Unknown	CSF: positive	Survive
	13	M	Unknown	CSF: positive	Survive
	11	F	Unknown	CSF: positive	Survive
Johswich KO, et al. 2012 [10]	13	M	Unknown	Blood: positive	Unknown
Zheng Xu, et al. 2015 [11]	7	F	Immunocompetent	Blood: positive	Survive
Ganesh K, et al. 2017 [12]	45–64^a^	M	Diabetes mellitus COPD	Pleural aspirate: positive	Survive
	15–24^a^	M	Unknown	CSF: positive	Survive
	5–9^a^	M	C6 deficiency	Blood: positive CSF: positive	Survive

*F* female, *M* male, *ALL* Acute lymphoblastic leukemia, *GVHD* graft versus host disease, *COPD* chronic obstructive pulmonary disease, *CSF* cerebrospinal fluid

^a^The age of three patients in reference [12] were described as age category

The laboratory data in our case revealed hypocomplementemia. At first, we suspected that hypocomplementemia appeared secondary due to sepsis [14]. However, because the patient’s vital signs did not reach the level of shock status [14], hypocomplementemia was considered to be secondary to the patient’s sole medical background, IgG4-related disease. Hypocomplementemia is a characteristic of IgG4-related disease; however, that does not suggest increased susceptibility to infection. Also, the patient had not taken immunosuppressive agents such as corticosteroids. We finally concluded that this was an accidental occurrence of IMD in this patient with IgG4-related disease. There remains the possibility that his job (taxi driver) was associated with the acquisition of the pathogen due to close contact with many persons. In a German study, 1.7% of healthy children and young adults possessed the non-capsulated *N. meningitidis* in their pharynx [6].

Based on a microbiological viewpoint of *N. meningitidis*, ST-11448 has a difference of only 1 nucleotide in the *fumC* gene (G at 207 to C) compared with those in ST-23 strain; however, this strain was uniquely recorded as ST-11448 in the MLST database at the time of Feburuary 2018 [15]. In addition, according to the Neisseria PorA and FetA typing database, [16, 17] 43 meningococcal strains were recorded as PorA VR1: 5 and FetA: F4-1, and among those, only one strain had been isolated in Sweden 2006, belonged to ST-23. On the other hand, a meningococcal strain listed as PorA VR2: 2-82 could not be found in the database. Taking the molecular epidemiological resolution of PorA and FetA typing into consideration, these microbiological results suggested that this *N. meningitidis* strain was a non-capsulated derivative of a domestic ST-23 strain rather than one imported from outside Japan.

In conclusion, this is the first documented case of IMD with non-typable *N. meningitidis* in Japan. Since the patient had no immunosuppressive drugs and the underlying disease was not considered to be an immunocompromising condition, we concluded that IMD had developed accidentally. This non-typable strain is considered to be low-pathogenic and few cases have been reported. We intend to collect more clinical cases that will allow us to establish the characteristics of IMD caused by this neglected pathogen.

## Abbreviations

IgG4: Iimmunoglobulin G4
IMD: Invasive Meningococcal Disease
